# The impact of long COVID on quality of life and work performance among healthcare workers in Portugal

**DOI:** 10.7717/peerj.19089

**Published:** 2025-03-10

**Authors:** Filipe Prazeres, Ana Paula Romualdo, Inês Campos Pinto, Joana Silva, Andreia M. Oliveira

**Affiliations:** 1 Family Health Unit Beira Ria, Gafanha da Nazaré, Portugal; 2 CINTESIS@RISE, MEDCIDS, Faculty of Medicine of the University of Porto, Porto, Portugal; 3 Faculty of Health Sciences, University of Beira Interior, Covilhã, Portugal; 4 Family Health Unit Ria Formosa, Faro, Portugal; 5 Family Health Unit Lendas d’Olhão, Olhão, Portugal

**Keywords:** Long COVID, Quality of life, Satisfaction with life, Work performance, Healthcare workers, Portugal

## Abstract

**Background:**

Coronavirus disease 2019 (COVID-19) is a multisystem infectious disease which affected 5.7 million people in Portugal. A subgroup of patients experienced long-term effects from the infection, now referred to as long COVID. Long COVID can considerably reduce the quality of life (QoL) of affected patients. This study aimed to evaluate the impact of long COVID on QoL and work performance among healthcare workers (HCWs) in Portugal.

**Methods:**

A cross-sectional correlational survey was performed in 348 HCWs employed either in hospitals, or non-hospital health facilities in Portugal. Participants completed an online survey using Google Forms between May and June 2024, which consisted of multiple-choice questions and took less than 10 min to fill out. Long COVID was considered present if the defined symptoms started at least 3 months after the primary infection of COVID-19, and persisted for at least 2 months. The outcome measures were performance at work and QoL. Performance at work was measured using a seven-point Likert scale and QoL was evaluated using the Satisfaction with Life Scale (SWLS). Data was analyzed using SPSS.

**Results:**

A total of 277 participants (79.6%) had history of SARS-CoV-2 infection, and 223 participants (64.1%) reported a history of long COVID. Extreme fatigue was reported by 158 participants (57.0%), cognitive dysfunction by 118 participants (42.6%), shortness of breath by 76 participants (27.4%), and persistent cough by 168 participants (60.6%). In the multivariate analysis, long COVID was significantly associated with lower SWLS scores indicating that long COVID negatively impacted QoL. Having two or more chronic diseases showed a trend towards lower performance, and extreme fatigue and cognitive dysfunction showed strong negative associations with performance.

**Discussion:**

Based on a national sample of HCWs (348 individuals), a high percentage of participants (64.1%) reported a history of long COVID. These results suggest that HCWs in Portugal have a prevalence of the disease similar to that of the worldwide population. Concerning performance at work, having two or more chronic diseases showed a trend towards lower performance, as well as extreme fatigue and cognitive dysfunction. Interestingly, we found a negative association between being a clinical secretary and SWLS. This might be explained by the specific challenges or stressors faced by clinical secretaries, which could negatively impact their QoL. In conclusion, long COVID was prevalent in the studied sample of HCWs and had a negative impact on their QoL. Extreme fatigue and cognitive dysfunction were strongly negatively associated with performance. This suggests the need for targeted care for HCWs as a group. The results of this study can guide healthcare authorities in addressing important long-term consequences that should be considered in rehabilitation programs for post-COVID-19 recovery.

## Introduction

Coronavirus disease 2019 (COVID-19) is a multisystem infectious disease caused by the severe acute respiratory disease coronavirus 2 (SARS-CoV-2) virus. It has affected 777 million people worldwide, with 5.7 million cases reported in Portugal as of November 10, 2024 (WHO, 2024). Most individuals returned to their baseline health status after the acute infection, while some experienced persistent health issues (Franco et al., 2022). A subgroup of patients experienced long-term effects from the infection, now referred to as long COVID, with reports of at least one sequela of COVID-19 in more than 80% of infected patients (Iqbal et al., 2021).

A World Health Organization (WHO)-endorsed Delphi consensus defined post-COVID-19 syndrome as, in simple terms, persistent symptoms and/or delayed or prolonged complications extending beyond 12 weeks that cannot be attributed to an alternative diagnosis (Soriano et al., 2022). Although its pathogenesis remains largely unknown, potential mechanisms include direct viral effects and prolonged inflammation with immune dysregulation (Nalbandian et al., 2021).

In a primary health care (PHC) study on non-hospitalized patients, more than half reported symptoms lasting at least 12 weeks after COVID-19, and had a positive correlation between the number of acute-phase symptoms and a higher risk of developing long COVID (Oliveira et al., 2023). In a recent systematic review and meta-analysis, long COVID symptoms include reported issues in mental health, gastrointestinal, cardiopulmonary, neurological, and pain-related areas (Natarajan et al., 2023). Moreover, an increased number of symptoms from both acute and long COVID have a significant negative impact on perceived overall quality of life (QoL) (Oliveira et al., 2023; Poudel et al., 2021).

The fact that long COVID can considerably reduce the QoL of affected patients emphasizes the need to understand its impact, and an increasing number of studies on long COVID have been published about this issue (Sugiyama et al., 2022). Nonetheless, there is limited evidence about how long COVID affects an individual’s cognitive evaluation of their quality of life, in particular life satisfaction. There is evidence that life satisfaction during the COVID-19 pandemic in 2021 showed a decline compared to levels recorded before the pandemic in 2019 (Phulkerd et al., 2023).

Work capacity is a dynamic process in which individuals fulfill the requirements of their tasks, using physical and mental abilities according to health conditions. Factors such as lifestyle, social characteristics, and work requirements significantly affect this process (Martinez, Latorre Mdo & Fischer, 2010). The SARS-CoV-2 pandemic created new challenges for occupational health, mainly related to the return to work of those affected by long-term symptoms, alongside decreased QoL, increased dependence on others for personal care, and impaired performance in daily activities (do Prado et al., 2022).

A study conducted in Cairo assessing the impact of long COVID on work performance among healthcare workers (HCWs) concluded that work performance was affected, with significantly higher functional limitations, but without increased absenteeism (Fouad, Zawilla & Maged, 2023). Data was collected using a self-reported standardized questionnaire, which may be subject to recall bias and personal over or underestimation (Fouad, Zawilla & Maged, 2023). On the other hand, a study in Germany identified that social, educational, and healthcare workers had considerably higher odds of sick leave, which can have deleterious effects not only on the health sector, but on the community (Schmachtenberg et al., 2023).

The present study aimed to evaluate the impact of long COVID on QoL and work performance among healthcare workers in Portugal.

## Materials and Methods

The sample for this cross-sectional correlational survey included HCWs employed either in hospitals or non-hospital health facilities in Portugal. Ethics approval was obtained from the University of Beira Interior ethics board (No. CE-UBI-Pj-2024-032-ID2266) before recruitment began, and the study adhered to the Declaration of Helsinki ethical standards. Participants completed an electronic questionnaire between May and June 2024. Recruitment was conducted through the social networking service Viva Engage (formerly Yammer) of the National Health Service and *via* the research team’s professional contacts. Participants were not compensated for their involvement. Electronic consent was obtained from all participants, and responses were anonymous.

A minimum sample size between 196 and 384 HCWs was calculated for proportions and considering the most conservative scenario (a proportion of 50%), a population of 174592 HCWs in Portugal (PORDATA, 2024), a level of confidence of 95% and an error margin of 5% to 7%. The sample size was calculated using the formula: $\rm n = [(1.96)^2 \times P \times (1-P)]/d^2$. Where n is the sample size, P is the expected prevalence, and d is margin of error or precision, as recommended by Sadiq et al. (2024) for cross-sectional studies.

Potential participants were invited to complete the survey online using Google Forms. One reminder was sent. The questionnaire, which consisted of multiple-choice questions and took less than 10 min to fill out, was pretested by 15 HCWs, who indicated that they understood all the questions.

Sociodemographic data, including gender, age, marital status, educational level, profession, and place of work, were collected. Data on clinical aspects were also gathered, including diagnosis of SARS-CoV-2, COVID-19 vaccination status, COVID-19 treated at home or hospital, and personal history of chronic disease(s). Long COVID was considered to be present if the HCWs experienced any of the following symptoms: extreme fatigue that significantly impaired functioning; cognitive dysfunction such as “brain fog,”, loss of concentration, or memory problems; shortness of breath; or a persistent cough. These symptoms must have lasted for at least 2 months and appeared at least 3 months after contracting SARS-CoV-2—even if they fluctuated—and there should be no other underlying condition causing them (such as hypertension, diabetes, *etc*.), in accordance with the WHO guidelines (WHO, 2021).

The outcome measures were performance at work and QoL.

Performance at work was measured using a seven-point Likert scale with the question: “In general, how do you perform your duties at work (compared to the period before having COVID-19)?” The scale ranged from 1:extremely difficult to 7:extremely easy.

QoL was evaluated using the Satisfaction With Life Scale (SWLS) (Diener et al., 1985), which has been widely used as a subjective measure of QoL (Pavot, 2014). SWLS consists of five items rated on a seven-point Likert scale, ranging from 1:totally disagree to 7:totally agree. This scale has been translated and validated for Portuguese in previous studies (Laranjeira, 2009; Neto, 1993), and is extensively used. A total score is obtained by summing the five items (ranging from 5 to 35 points), with higher scores indicating higher levels of perceived QoL. Cronbach’s alpha for the scale was originally reported as 0.87 (Diener et al., 1985), and was 0.89 for the current sample.

Data was analysed with SPSS, version 29.0 (IBM Corporation, 2023). For descriptive statistics, means and standard deviations, frequencies and percentages are presented. SWLS reliability was measured with Cronbach’s alpha, considering good reliability for alpha > 0.70. Generalized linear models were implemented to measure the association of explanatory variables with outcomes, namely SWLS and HCWs’ performance at work. Coefficients were estimated with maximum likelihood function. All potential explanatory variables were associated with outcomes, first with univariate models. All variables with *p*-value < 0.10 were then included in a multivariate model. Significance of multivariate models was deemed for *p* < 0.05.

During the preparation of this manuscript the authors used ChatGPT-4o in order to improve the language of the manuscript and correct grammatical errors. After using this tool, the authors reviewed and edited the content as needed and take full responsibility for the content of the publication.

## Results

Table 1 presents HCWs’ characteristics. Mean age was 48.0 years (SD = 11.0), ranging from 21 to 73. There were 50 male (14.4%) and 298 female (85.6%) participants. Concerning marital status, 122 (35.1%) participants had no partner, while 226 (64.9%) had a partner. Educational levels included 76 participants (21.8%) with 9 to 12 years of education, 138 participants (39.7%) with a university degree, and 134 participants (38.5%) with a Masters degree or a PhD. The professions represented in the sample included 67 medical doctors (19.3%), 123 nurses (35.3%), 100 clinical secretaries (28.7%), and 58 health or diagnostic and therapeutic technicians (16.7%). Participants’ place of work was in hospitals (45.4%) and in non-hospital healthcare facilities (54.6%). Regarding the history of chronic diseases, 170 participants (48.9%) reported no diseases, 106 participants (30.5%) reported one disease, and 72 participants (20.7%) reported two or more diseases.

**Table 1:** Main characteristics of the study participants.

Variable	
Age	48.0 (11.0)
Sex	
Male	50 (14.4%)
Female	298 (85.6%)
Marital status	
Without partner	122 (35.1%)
With partner	226 (64.9%)
Education	
9 to12 years	76 (21.8%)
University degree	138 (39.7%)
Masters degree or PhD	134 (38.5%)
Profession	
Medical doctor	67 (19.3%)
Nurse	123 (35.3%)
Clinical secretary	100 (28.7%)
Health or diagnostic and therapeutic technician	58 (16.7%)
Place of work	
Hospital	158 (45.4%)
Non-hospital healthcare facility	190 (54.6%)
History of chronic diseases	
No diseases	170 (48.9%)
1 disease	106 (30.5%)
2 or more diseases	72 (20.7%)

**Note:**

Results presented as *n* (%) for categorical variables and Mean (SD) for age.

Table 2 presents the results related to SARS-CoV-2 variables. Concerning participants’ vaccination status, 71 (20.4%) had no history of SARS-CoV-2 infection, 55 (15.8%) were not vaccinated, 35 (10.1%) did not complete the full vaccination program, and 187 (53.7%) completed the vaccination program.

**Table 2:** SARS-CoV-2 related variables.

	*n*	%
SARS-CoV-2 vaccination program		
No history of SARS-CoV-2 infection	71	20.4%
No vaccine	55	15.8%
Incomplete vaccination program	35	10.1%
Complete vaccination program	187	53.7%
History of SARS-CoV-2 infection	277	79.6%
History of long COVID	223	64.1%
Extreme fatigue	158	57.0%
Cognitive dysfunction	118	42.6%
Shortness of breath	76	27.4%
Cough	168	60.6%

A total of 277 participants (79.6%) had a history of SARS-CoV-2 infection. A history of long COVID was reported by 223 participants (64.1%). Extreme fatigue was reported by 158 participants (57.0%), cognitive dysfunction by 118 participants (42.6%), shortness of breath by 76 participants (27.4%), and persistent cough by 168 participants (60.6%).

Table 3 presents the means and standard deviations for the studied outcomes. HCWs’ performance at work had a mean score of 5.00 (SD 1.60). For the Satisfaction with Life Scale (SWLS), the total score was 22.97 (SD 6.05).

**Table 3:** Means (M) and standard deviations (SD) for HCWs’ performance at work and SWLS.

	M	SD
In general, how do you perform at your workplace [1–7]	5.00	1.60
The Satisfaction with Life Scale (SWLS) [1–7]		
1. In most ways my life is close to my ideal	4.71	1.40
2. The conditions of my life are excellent	4.52	1.32
3. I am satisfied with my life	4.81	1.35
4. So far, I have gotten the important things I want in life	4.97	1.42
5. If I could live my life over, I would change almost nothing	3.95	1.71
SWLS total (Cronbach’s alpha of the five items = 0.893) [5–35]	22.97	6.05

Next, we present the results for univariate and multivariate generalized linear model associations with SWLS and performance at work (Table 4). Concerning SWLS, in the univariate analysis, long COVID was associated with a decrease in SWLS scores (B = −1.25, SE = 0.67, *p* = 0.062), with a marginal association at the 0.10 level. Age and female sex did not show significant associations with SWLS scores (B = −0.03, SE = 0.03, *p* = 0.295; B = −0.90, SE = 0.92, *p* = 0.327, respectively). Higher education was associated with increased SWLS scores, with a Master or PhD showing a significant positive association (B = 2.72, SE = 0.85, *p* = 0.001). Compared to medical doctors, clinical secretaries had significantly lower SWLS scores (B = −3.45, SE = 0.94, *p* < 0.001), and nurses also showed a trend towards lower scores (B = −1.54, SE = 0.90, *p* = 0.087). Non-hospital healthcare facility workers did not differ significantly from hospital workers (B = 0.69, SE = 0.65, *p* = 0.288). Participants with two or more chronic diseases had lower SWLS scores (B = −1.65, SE = 0.84, *p* = 0.050).

**Table 4:** Uni and multivariate generalized linear model associations with SWLS.

	Univariate		Multivariate		
	B (SE)	*p*-value	B (SE)	*p*-value	95% CI
Long COVID	−1.25 (0.67)	0.062^‡^	−1.35 (0.68)	0.048*	[−2.68 to −0.01]
Age	−0.03 (0.03)	0.295	–	–	–
Female	−0.90 (0.92)	0.327	–	–	–
Education					
9 to12 years	REF	REF	REF	REF	REF
University degree	1.19 (0.85)	0.161	−0.33 (1.11)	0.767	[−2.49 to 1.84]
Masters degree or PhD	2.72 (0.85)	0.001**	0.76 (1.26)	0.546	[−1.71 to 3.23]
Profession					
Medical Doctor	REF	REF	REF	REF	REF
Nurse	−1.54 (0.90)	0.087^‡^	−1.04 (0.91)	0.253	[−2.81 to 0.74]
Clinical secretary	−3.45 (0.94)	<0.001***	−2.86 (1.25)	0.023*	[−5.32 to 0.40]
Health or diagnostic and therapeutic technician	−1.66 (1.06)	0.119	−1.21 (1.09)	0.266	[−3.34 to 0.92]
Place of work					
Hospital	REF	REF	REF	REF	REF
Non-hospital healthcare facility	0.69 (0.65)	0.288	–	–	–
History of chronic diseases					
No diseases	REF	REF	REF	REF	REF
1 disease	0.01 (0.74)	0.994	0.50 (0.75)	0.509	[−0.97 to 1.96]
2 or more diseases	−1.65 (0.84)	0.050^‡^	−1.46 (0.83)	0.079^‡^	[−3.09 to 0.17]

**Notes:**

Results presented as unstandardized coefficients (B), standard errors (SE) and 95% confidence intervals (CI).

‡ *p* < 0.10.

* *p* < 0.05.

** *p* < 0.05.

*** *p* < 0.001.

*n* = 348.

In the multivariate analysis, long COVID was significantly associated with lower SWLS scores (B = −1.35, SE = 0.68, *p* = 0.048, 95% CI [−2.68 to −0.01]), indicating that long COVID negatively impacted QoL. Higher education did not maintain a significant association in the multivariate model (university degree: B = −0.33, SE = 1.11, *p* = 0.767; Master or PhD: B = 0.76, SE = 1.26, *p* = 0.546). The negative association between being a clinical secretary and SWLS scores remained significant (B = −2.86, SE = 1.25, *p* = 0.023, 95% CI [−5.32 to −0.40]). Other professional roles and the number of chronic diseases did not show significant associations in the multivariate model.

Regarding performance in the workplace (Table 5), in the univariate analysis, long COVID was significantly associated with decreased workplace performance (B = −0.83, SE = 0.23, *p* < 0.001). Age had a negative association with performance (B = −0.02, SE = 0.09, *p* = 0.049), and female sex showed a trend towards lower performance (B = −0.53, SE = 0.27, *p* = 0.050). Higher education, specifically having a Master or PhD, was positively associated with performance (B = 0.49, SE = 0.25, *p* = 0.047). Nurses and clinical secretaries showed lower performance compared to medical doctors (B = −0.53, SE = 0.26, *p* = 0.039; B = −0.69, SE = 0.27, *p* = 0.012, respectively). Those with one chronic disease had lower performance (B = −0.53, SE = 0.21, *p* = 0.010), and those with two or more diseases had even lower performance (B = −0.99, SE = 0.24, *p* < 0.001). Extreme fatigue (B = −1.37, SE = 0.17, *p* < 0.001), cognitive dysfunction (B = −1.28, SE = 0.11, *p* < 0.001), and shortness of breath (B = −0.80, SE = 0.20, *p* < 0.001) were all associated with significantly decreased workplace performance.

**Table 5:** Uni and multivariate generalized linear model associations with HCWs’ performance at work.

	Univariate		Multivariate		
	B (SE)	*p*-value	B (SE)	*p*-value	95% CI
Long COVID	−0.83 (0.23)	<0.001***	(a)	(a)	(a)
Age	−0.02 (0.09)	0.049*	−0.01 (0.01)	0.411	[−0.02 to 0.01]
Female	−0.53 (0.27)	0.050^‡^	−0.21 (0.24)	0.383	[−0.67 to 0.26]
Education					
9 to12 years	REF	REF	REF	REF	REF
University degree	0.14 (0.25)	0.589	0.05 (0.30)	0.872	[−0.53 to 0.63]
Masters degree or PhD	0.49 (0.25)	0.047*	0.33 (0.33)	0.321	[−0.32 to 0.97]
Profession					
Medical Doctor	REF	REF	REF	REF	REF
Nurse	−0.53 (0.26)	0.039*	−0.02 (0.23)	0.930	[−0.47 to 0.43]
Clinical secretary	−0.69 (0.27)	0.012*	0.04 (0.33)	0.903	[−0.62 to 0.70]
Health or diagnostic and therapeutic technician	−0.43 (0.30)	0.154	0.04 (0.28)	0.894	[−0.50 to 0.58]
Place of work					
Hospital	REF	REF	REF	REF	REF
Non-hospital healthcare facility	−0.08 (0.19)	0.665	–	–	–
History of chronic diseases					
No diseases	REF	REF	REF	REF	REF
1 disease	−0.53 (0.21)	0.010*	−0.11 (0.19)	0.545	[−0.48 to 0.25]
2 or more diseases	−0.99 (0.24)	<0.001***	−0.44 (0.23)	0.054^‡^	[−0.88 to 0.01]
Extreme fatigue	−1.37 (0.17)	<0.001***	−0.86 (0.19)	<0.001***	[−1.24 to −0.49]
Cognitive dysfunction	−1.28 (0.11)	<0.001***	−0.83 (0.19)	<0.001***	[−1.19 to −0.46]
Shortness of breath	−0.80 (0.20)	<0.001***	−0.07 (0.20)	0.734	[−0.47 to 0.33]
Cough	−0.21 (0.19)	0.270	–	–	–

**Notes:**

Results presented as unstandardized coefficients (B), standard errors (SE) and 95% confidence intervals (CI).

‡ *p* < 0.10.

* *p* < 0.05.

*** *p* < 0.001.

*n* = 277 (excluded the 71 participants with no SARS-CoV-2 infection).

(a) Removed due to strong association with long COVID symptoms.

In the multivariate analysis, long COVID was removed due to strong association with long COVID symptoms. Age and female sex were no longer significant (B = −0.01, SE = 0.01, *p* = 0.411; B = −0.21, SE = 0.24, *p* = 0.383, respectively). Higher education did not maintain significant associations with workplace performance (university degree: B = 0.05, SE = 0.30, *p* = 0.872; Master or PhD: B = 0.33, SE = 0.33, *p* = 0.321). Professionally, nurses and clinical secretaries did not show significant associations in the multivariate model (B = −0.02, SE = 0.23, *p* = 0.930; B = 0.04, SE = 0.33, *p* = 0.903). Having two or more chronic diseases showed a trend towards lower workplace performance (B = −0.44, SE = 0.23, *p* = 0.054). Extreme fatigue and cognitive dysfunction continued to show strong negative associations with performance (B = −0.86, SE = 0.19, *p* < 0.001, 95% CI [−1.24 to −0.49]; B = −0.83, SE = 0.19, *p* < 0.001, 95% CI [−1.19 to −0.46]). Shortness of breath was not significant in the multivariate analysis (B = −0.07, SE = 0.20, *p* = 0.734).

## Discussion

Long COVID is currently considered a multisystemic disease, having a tremendous impact on the health-related QoL affecting 31–69% of the worldwide population after acute SARS-CoV-2 infection (Groff et al., 2021; Koc et al., 2022; Turner et al., 2023). Therefore, this contributes considerably to the overall burden of disease and reduced satisfaction with life (Turner et al., 2023).

In the present study, we aimed to evaluate the impact of long COVID on QoL and work performance among HCWs in Portugal from May to July of 2024.

Based on a national sample of HCWs (348 individuals), a high percentage of participants (64.1%) reported a history of long COVID, which is corroborated by other reports, such as the systematic review by Groff et al. (2021). The study results suggest that HCWs in Portugal have a prevalence of the disease similar to that of the worldwide population. However, since long COVID has various definitions in the literature, direct comparisons between the results of the present study with those of other studies are not possible. Considering that long COVID may be a disease of accelerated biological aging—affecting endothelial, adipose, and musculoskeletal tissues (Lauwers et al., 2022), the impact on HCWs can be significant, leading to increased risk of various health issues.

According to the spectrum of symptoms presented in the online questionnaire, the most common symptoms stated were persistent cough (60.6%), extreme fatigue (57.0%), cognitive dysfunction (42.6%) and shortness of breath (27.4%). Extreme fatigue, cognitive dysfunction and shortness of breath have frequently been reported as the most prevalent symptoms in previous studies (Pretorius et al., 2022). A study of healthcare workers found an increased prevalence of symptoms related to both the pandemic and post-COVID conditions. These symptoms, including fatigue, headache, insomnia, cognitive impairment, and stress/burnout, are associated with greater functional impairment, higher absenteeism, and a worsening quality of life (Nehme et al., 2022). However, cough has been described as a non-prevalent symptom of long COVID, stated by Turner et al. (2023) in a study based on an Italian population. This inconsistency between studies’ results may reflect characteristics of the COVID strain, the genetic profile of Portuguese HCWs, population habits such as smoking, or the use of different definitions of long COVID.

Recent studies suggest a significant link between autonomic nervous system dysfunction and post-COVID-19 symptoms, including fatigue, dizziness, dyspnea, syncope, heart palpitations, orthostatic intolerance, nausea, and vomiting (Jammoul et al., 2023). Proposed mechanisms for this dysfunction include direct viral invasion of autonomic centers, autoimmunity, persistent inflammation, and imbalance of the renin-angiotensin system (Jammoul et al., 2023). Thus, timely and accurate diagnosis, along with careful management, will be essential for the recovery of HCWs (Dani et al., 2021).

Regarding the results of the present study, long COVID was significantly associated with lower SWLS scores (B = −1.35, SE = 0.68, *p* = 0.048, 95% CI [−2.68 to −0.01]), indicating that long COVID negatively impacted QoL, in particular life satisfaction. These results are consistent with previous studies. In one study conducted with HCWs at a medical college hospital regarding health-related quality of life, 73.1% of respondents reported physical health issues, while 12.1% experienced limitations in usual activities due to health problems (Suresh et al., 2023). Another study, involving 766 participants with long COVID symptoms, identified poor work ability as the most significant factor contributing to low life satisfaction (Ekstrand et al., 2022).

Interestingly, we found a negative association between being a clinical secretary and SWLS scores. This might be explained by the specific challenges or stressors faced by clinical secretaries, which could negatively impact their quality of life. This finding highlights need for interventions that address their workplace conditions while also providing better support. Contrary to common healthcare stereotypes or popular belief, non-hospital healthcare facility workers did not differ significantly from hospital workers (B = 0.69, SE = 0.65, *p* = 0.288). This suggests that the work environment or role itself may not significantly affect life satisfaction, or that non-hospital workers face unique challenges that offset the perceived advantages.

Concerning performance at work, having two or more chronic diseases showed a trend towards lower performance (B = −0.44, SE = 0.23, *p* = 0.054). Extreme fatigue and cognitive dysfunction showed strong negative associations with performance (B = −0.86, SE = 0.19, *p* < 0.001, 95% CI [−1.24 to −0.49]; B = −0.83, SE = 0.19, *p* < 0.001, 95% CI [−1.19 to −0.46]). These results are in agreement with a study comprising an international cohort of 3,762 COVID patients, in which, 67.5% of the participants were unable to return to work or required a reduction in their work schedule (Davis et al., 2021). Unfortunately, the present study was not designed to quantify absences or reductions in work schedules among Portuguese HCWs due to long COVID, which may represent a limitation of this study.

Regarding other limitations of the present study, we note that reliance on self-reporting by HCWs may increase susceptibility to recall bias, and the sample size was relatively small. Self-selection bias and nonresponse bias may also be present, as HCWs who volunteered to participate might differ from those who did not. For example, symptomatic HCWs may have been more likely to complete the questionnaire, whereas those without COVID-19-related complaints might have been less inclined to participate. A larger sample size would likely yield more statistically robust results. Additionally, only 50 males responded to the survey, possibly due to culturally motivated behaviors regarding men’s concerns with their health. Nonetheless, more women work in healthcare than men, making the results of the present study relevant even taking into consideration the sample size. Interestingly, a meta-analysis compiling 41 studies concluded that being female was associated with an increased risk of developing long COVID (Tsampasian et al., 2023).

Additionally, the severity of the acute phase of the infection was not addressed in the online questionnaire in the present study, leading to a lack of information about its impact on long COVID. Furthermore, more detailed information regarding the specific chronic diseases reported by participants would help clarify the association between long COVID and common chronic conditions.

Also, future studies on long COVID will benefit from analyzing the potential protective role of SARS-CoV-2 antibodies, particularly the association of antibody levels with age and time since the last vaccine dose, as well as the emergence of new variants—factors that a self-administered questionnaire could not explore.

The strengths of the study include a national sample of 348 participants and the use of the WHO’s definition of post-COVID-19 condition to define long COVID. Another strength was adjusting our analysis for age, gender, number of comorbidities, profession, and level of education, which contributed to providing comprehensive information.

## Conclusions

Long COVID was prevalent in the studied sample of HCWs and negatively impacted their QoL. Extreme fatigue and cognitive dysfunction were strongly and negatively associated with workplace performance. This indicates that there is a need for targeted care for HCWs as a group. The results of this study can guide healthcare authorities in addressing important long-term consequences that should be considered in rehabilitation programs for post-COVID-19 recovery.

## Supplemental Information

10.7717/peerj.19089/supp-1 Supplemental Information 1 SPSS data file.

## Additional Information and Declarations

### Data Availability

The following information was supplied regarding data availability:

The raw data are available as a Supplemental File.
